# Acceptability, Feasibility, and Preliminary Efficacy of *Schools Championing Safe South Africa*, a Social Norms Intervention to Prevent HIV Risk Behavior and Perpetration of Intimate Partner Violence Among Teenage Boys

**DOI:** 10.1007/s10461-025-04723-w

**Published:** 2025-04-22

**Authors:** Caroline Kuo, Abigail Harrison, Lindsay M. Orchowski, Yandisa Sikweyiya, Alan Berkowitz, Haley Adrian, Nandipha Gana, Akhona Rasmeni, Tracy McClinton Appollis, Portia Nevhungoni, Catherine Mathews

**Affiliations:** 1 Department of Health Studies, College of Arts and Sciences, American University, 4400 Massachusetts Avenue, NW, Washington, DC 20016 USA; 2 Department of Psychiatry and Mental Health, University of Cape Town, Cape Town, South Africa; 3 Rhode Island Hospital, Providence, RI USA; 4 Department of Behavioral and Social Sciences, Brown University School of Public Health, Providence, RI USA; 5 Gender and Health Research Unit, South African Medical Research Council, Pretoria, South Africa; 6 Mount Shasta, CA USA; 7 Health Systems Research Unit, South African Medical Research Council, Cape Town, South Africa; 8 Biostatistics Unit, South African Medical Research Council, Cape Town, South Africa; 9 School of Public Health, University of the Witwatersrand, Johannesburg, South Africa

**Keywords:** HIV, Intimate partner violence, Bystander behavior, HIV risk, Prevention, South Africa

## Abstract

*Schools Championing Safe South Africa* is an intervention to prevent sexual violence perpetration and HIV/STI risk behavior among teenage boys, focusing on correcting misperceived social norms regarding risk behavior and engaging boys, teachers and peers in school. We tested its acceptability, feasibility, and preliminary efficacy in a pilot RCT (*N* = 282). 99% of intervention boys reported high satisfaction with content, format, and delivery. There was good facilitator fidelity to the manualized protocol and 99% retention at 6-month follow-up. Among intervention boys, completed acts of any sexual violence perpetration (touching, oral, anal, and/or vaginal sex) decreased from 71% (95% CI: 61%, 80%) at baseline to 55% (95% CI: 44%, 66%) at 1 month follow-up, with a percentage difference of 15% (95% CI: 4%, 26%; *p* = 0.004). At 6 months, change was not significant (72–68%; *p* = 0.353). For the intervention group, attempted acts of any sexual violence perpetration reduced from 49% (95% CI: 40%, 58%) at baseline to 25% (95% CI: 17%, 33%) at 1 month, with a percentage difference of 22% (95% CI: 11%, 32%; *p* < 0.001) but was not sustained at 6 months (47–43%; *p* = 0.446). Across timepoints, the control group did not show significant changes in completed or attempted perpetration. There were no significant changes in condom use in the intervention or control groups. Behavioral signals of positive change for prevention of sexual violence perpetration combined with high acceptability and feasibility indicate that the intervention should be tested further for efficacy.

## Introduction

HIV and sexual violence are synergistic epidemics. Several systematic reviews and meta-analyses identify causal and non-causal mechanisms linking the epidemics of HIV and sexual violence. Causal mechanisms linking increased HIV risk with sexual violence include increased genital or anal tissue trauma associated with increased infection risk [1]. Non-causal mechanisms include positive correlation between HIV infection and those who perpetrate intimate partner violence (IPV) including sexual violence [2] and higher rates of HIV risk behaviors among IPV perpetrators including decreased condom use [3, 4], concurrent and/or multiple sexual partners [3, 5], alcohol and substance use, and higher rates of HIV and other sexually transmitted infections (STIs) [6, 7]. HIV acquisition risk is significantly higher among individuals who have experienced IPV [8]. For example, a global systematic review and meta-analysis of 28 studies with *N* = 331,468 women (including 4 South African studies), indicated that any type of IPV was significantly associated with HIV infection [9]. 

South Africa is the ideal geographic site to develop prevention science for the synergistic epidemics of IPV and HIV. South Africa has the largest HIV epidemic of any country in the world [10], with adolescents accounting for the majority of new HIV infections [11]. Adolescents are naturally at increased risk for HIV due to normal developmental milestones [12]. Initial sexual experiences frequently occur at this age, corresponding to increased risk for acquisition of HIV, other STIs, and IPV. National data in South Africa show that adolescent boys are engaging in HIV risk behavior at higher rates than girls: 67.7% of boys reported condomless last sex compared to 49.8% for girls, and 25.5% of boys reported two or more sexual partners compared to 9% among girls [13]. South Africa also has a high global burden of IPV. Globally, the sub-Saharan African region, including South Africa, has the highest prevalence for both intimate partner and non-partner sexual and/or physical violence at 65.6% (95% CI: 53.6–77.7%) [14] and 21% (95% CI: 4.5–37.5%) respectively [15]. While violence occurs across the lifespan, preventive interventions are urgently needed during adolescence and among boys. This is because the vast majority of sexual violence is perpetrated by boys and men in South Africa, with a survey in South Africa reporting that 1 in 3 men (31.9%) reported rape perpetration [16]. In South Africa, the ideal age range for targeting adolescent boys most at risk for HIV and sexual violence perpetration is age 15 to 17 years. In a large longitudinal prospective cohort study of South African adolescents, the median age of penetrative sexual debut was 15 years, with 38.2% of boys engaged in penetrative debut at this age; [17] and in a large representative sample of rape perpetration among men in South Africa, participants reported an average age of 17 years for first rape perpetration [18, 19]. 

There is limited evidence for adolescent-tailored HIV-IPV interventions. A recently published systematic review on integrated HIV and IPV interventions for adolescents in sub-Saharan Africa identified only six interventions that concomitantly address HIV and IPV among adolescents [20]. Four interventions occurred in South Africa: *PREPARE*,* IMAGE*,* Stepping Stones*, and Kalichman’s unnamed intervention. Only Kalichman’s intervention for both HIV and IPV exclusively targeted IPV perpetration (rather than victimization) and was male-focused but did not include adolescents under 18 years; the intervention group did not show any differences for increased condom use, decreased number of sexual partners, or decreased occurrence of unprotected sex. At the six-month follow-up, the intervention group showed decreased perpetration of past-month physical IPV (OR 0.3, 95% CI 0.2–0.4) [21]. The other three interventions in South Africa either examined HIV alongside violence as targets of the interventions, and only two interventions (*PREPARE* and *Stepping Stones*) specifically included adolescents [20]. In *PREPARE*, a cluster RCT in 42 schools, targeting all genders and a younger age range (13–14 years) than our intervention age group, intervention participants were less likely to report IPV victimization (35.1 vs. 40.9%; OR: 0.77; 95% CI 0.61–0.99) but had no changes in HIV risk [22]. The *IMAGE* RCT included 14–35 year olds, all girls and women, and investigated whether a microfinance intervention delivered over 6–9 months providing economic stability and complemented with education on male gender norms, domestic violence, sexuality and HIV could reduce IPV among adults. After two years, intervention participants reported lower risk of past-year physical or sexual violence by an intimate partner (adjusted risk ratio = 0.45; 95% CI: 0.23–0.91) but no changes in HIV incidence [23]. *Stepping Stones* RCT was a participatory intervention designed to reduce HIV, herpes simplex type 2 (HSV-2) among young men and women 15–26 years old living in the Eastern Cape province of South Africa. The group-based intervention was delivered in weekly sessions for 6–8 weeks. *Stepping Stones* was not associated with reduced HIV incidence, but demonstrated 33% reduction in Type 2 herpes simplex virus (aIRR 0.67, 95% CI 0.46–0.97). A lower proportion of male participants reported IPV perpetration at two-year follow-up in the intervention group compared to the control (adjusted odds ratio [aOR] 0.62, 95% CI 0.38–1.01) [24]. The evidence from the review further underscores the need for interventions tailored for adolescents, targeting the school environment, and focusing on primary prevention of violence perpetration integrated with HIV.

None of the existing HIV-IPV intervention studies in sub-Saharan Africa described above utilized a social norms approach as the main pathway for intervention change for integrated prevention of HIV and IPV [20]. The social norms approach is ideally suited for the developmental stage of adolescence in well-defined communities such as schools. Early experiences around relationships, sexual behavior, and violence during adolescence can uniquely shape young people’s social norms around what are healthy/unhealthy norms for future relationships [25]. Schools constitute important community environments for social norms formation; adolescents spend a large segment of their day in school and social interactions with student peers and teachers are critical to identity development and health behaviors [26–30]. Thus, leveraging the school ecology (including teachers and student peers) to establish healthy norms among adolescents just as they are establishing attitudes towards sex, relationships, and violence may help to shape patterns of healthy behavior across the lifespan. Social norms are differentiated from personal attitudes in that they convey ideas about behavior that are (mis)perceived to be normal and socially accepted by one’s peers [31]. 

A specific approach to addressing social norms—correcting misperceived social norms—has not yet been tested for integrated primary prevention of HIV-IPV. Yet, addressing social norms has shown promise in preventing bullying, problem alcohol use, and dating violence, as well as mosquito nets for malaria prevention in Southern Africa [32–35]. Correcting misperceived social norms has the potential to address powerful risk and protective pathways for interlinked risks of HIV and IPV. Changing social norms is an important approach, but is not sufficient by itself and must be combined with behavioral change as well [36]. Despite a promising body of research supporting the application of the social norms approach to IPV, further evidence is needed to understand its applicability to integrated prevention of HIV and violence. Experimental evidence is needed to establish the effects of how a social norms approach might work with different sub-groups and with the involvement of different entry points into their social ecology. In light of the above, our intervention approach integrates prevention of both HIV risk and sexual violence perpetration, with a purposive focus on adolescent boys by correcting misperceived social norms combined with behavior change. In this paper, we present results on the feasibility, acceptability, fidelity, and preliminary efficacy of an intervention for preventing risk behavior related to acquisition of HIV and intimate partner violence perpetration among teenage boys in South Africa that uses a social norms approach. The intervention focuses on the target population of boys themselves, as well as engaging peers and teachers in the boys’ school environment in prevention of HIV risk behavior and violence. Results are reported following CONSORT guidelines for quality reporting on randomized controlled trial study designs [37]. 

## Intervention

The *Schools Championing Safe South Africa* intervention engages the teachers and student peers of teenage boys in the process of supporting the target population—teenage boys—in prevention of HIV risk behavior and intimate partner violence perpetration. Following guidelines for interventions using the social norms approach [38], the basis of the intervention content is derived from school-specific data of study participants; the specificity of the data to the school community of the target population (teenage boys) is vital to how the intervention works since data directly relates to their perceptions around behaviors/norms, and their peer and teacher perceptions around behaviors/norms about HIV/STI risk behaviors, IPV and bystander intervention. To generate the normative data used in the intervention content, we completed a social ecology survey, focusing on coverage of students in grades 10–11 where most of the age range of target participants (15–17 year olds) are located, as well as teachers. Interested and eligible adolescents with parental consent and who gave under-age assent were invited to complete a brief social ecological survey assessing three areas. First, we gathered data on self-reported risk behavior related to IPV and HIV (students only). An example of this data included comparing actual rates of condom use with perceived rates of condom use among school peers. Second, we gathered data on the perceived prevalence of IPV and sexual behaviors among students in their school. An example of this data included comparing actual rates of perpetration of sexual violence with perceived rates of student peers engaging in sexual violence. Third, we gathered data on perceived peer norms and attitudes around sex, HIV, gender, and IPV. An example of this data included comparing actual gender equitable norms versus perceived norms from student peers around gender equitable norms. Survey responses were anonymous, and adolescents used electronic tablets to diminish response bias that might occur via interviewer-administered surveys. Analyses focused on comparing actual norms and behaviors versus perceived norms and behaviors of peers. For example, if 83% of participants reported that they would believe someone who was sexually assaulted, and these same participants reported that they perceived that only 49% of their peers would believe someone who said they were sexually assaulted, the analyses would evaluate whether there was a misalignment between actual norms/behaviors and perceived norms/behaviors. We defined significant misalignment as a difference of ≥ 20% between actual norms/behaviors and perceived peer norms/behaviors. In the prior example, there was significant misalignment between those who would believe someone if they said they were sexually assaulted versus those the perception of whether peers would believe someone who said they were sexually assaulted (83% − 49% = 34%). The data from the survey of social ecology was then fed into the intervention content if exposing the mis-alignment data would “nudge” participants towards preventive behavior (i.e., exposing that the actual percentage of participants would believe someone who said they were sexually assaulted was much larger than what was perceived). To further refine intervention content, we also included a qualitative intervention refinement phase where a separate school, very similar to the two schools participating in the pilot RCT, had teachers and teenagers give feedback on the draft materials. During the qualitative refinement stage, we showed draft posters to this group of participants and elicited feedback on the clarity of messaging, the visual congruency of the poster with the school communities we would be working in, and other feedback on whether the information could be improved in regard to acceptability. Notable was that during this phase, adolescents and teachers in schools with similar population characteristics found the content to be contextually and culturally appropriate.

The intervention was delivered in two parts: (1) a social norms marketing poster campaign and (2) behavioral change lessons that were theoretically derived from two complementary theoretical foundations. *Schools Championing Safe South Africa’s* HIV risk prevention components are based on Information-Motivation-Behavioral (IMB) theory [39] which theorizes that behavior change is most effective when preventive information (rates of HIV, pathways to HIV infection, etc.) is combined with motivation to change (impact of HIV on futures, etc.) and with behavioral practice to build confidence (correct use of condoms, etc.) [39]. The change strategy of the intervention’s HIV components included: (1) providing information to increase HIV knowledge around preventive and protective behaviors, (2) motivating teenagers to implement preventive and protective behaviors and (3) increasing protective behaviors by practicing these behaviors as building self-efficacy for prevention behaviors (condom use, partner consent and safe sex negotiation and healthy sexual relationships) [40–42]. *Schools Championing Safe South Africa’s* violence prevention components were based on the Integrated Model of Sexual Assault and Acquaintance Rape [43, 44]. This conceptual model suggests that interventions to prevent violence, especially intimate partner violence, are most salient when targeting risk and protective factors across the social ecology (such as school communities) including social norms. The change strategies of the violence prevention components of the intervention included: (1) understanding conditions of sexual consent; (2) increasing male empathy regarding the effects of sexual violence including intimate and interpersonal violence, and sexual aggression; (3) correcting misperceptions regarding sexual violence prevalence as well as prevalence of sex, and consequences of these misperceptions; (4) increasing use of bystander strategies; and (5) increasing awareness of risk for sexual violence and aggressive behavior and links to HIV transmission. The third component on correcting misperceived social norms that support perpetration of violence or risk behaviors relating to HIV/STI transmission is especially critical; our approach incorporated a “norms correction” strategy. This approach recognizes the importance of peer influence on people’s health behavior. Specifically, it recognizes that healthy attitudes and behaviors may be underestimated, while unhealthy attitudes and behavior may be overestimated. What makes this particular social norms intervention distinct from other social norms approaches is (1) that we used real time data from a well-defined community that mattered to target person (in this case schools) and (2) we exposed the “good news” when we found misalignments between actual and perceived norms: where the healthy norms were underestimated and negative norms aligned to HIV or IPV perpetration were overestimated. We exposed misalignments and in doing so, we intended to correct or reduce misperceptions related to harmful behaviors while also increasing prosocial behaviors. Exposing the real “good news” about the prevalence of healthy social norms related to IPV and HIV can nudge people towards that positive behavior. This conceptual model has guided other successful preventive interventions in settings other than South Africa [45]. 

**Fig. 1:**
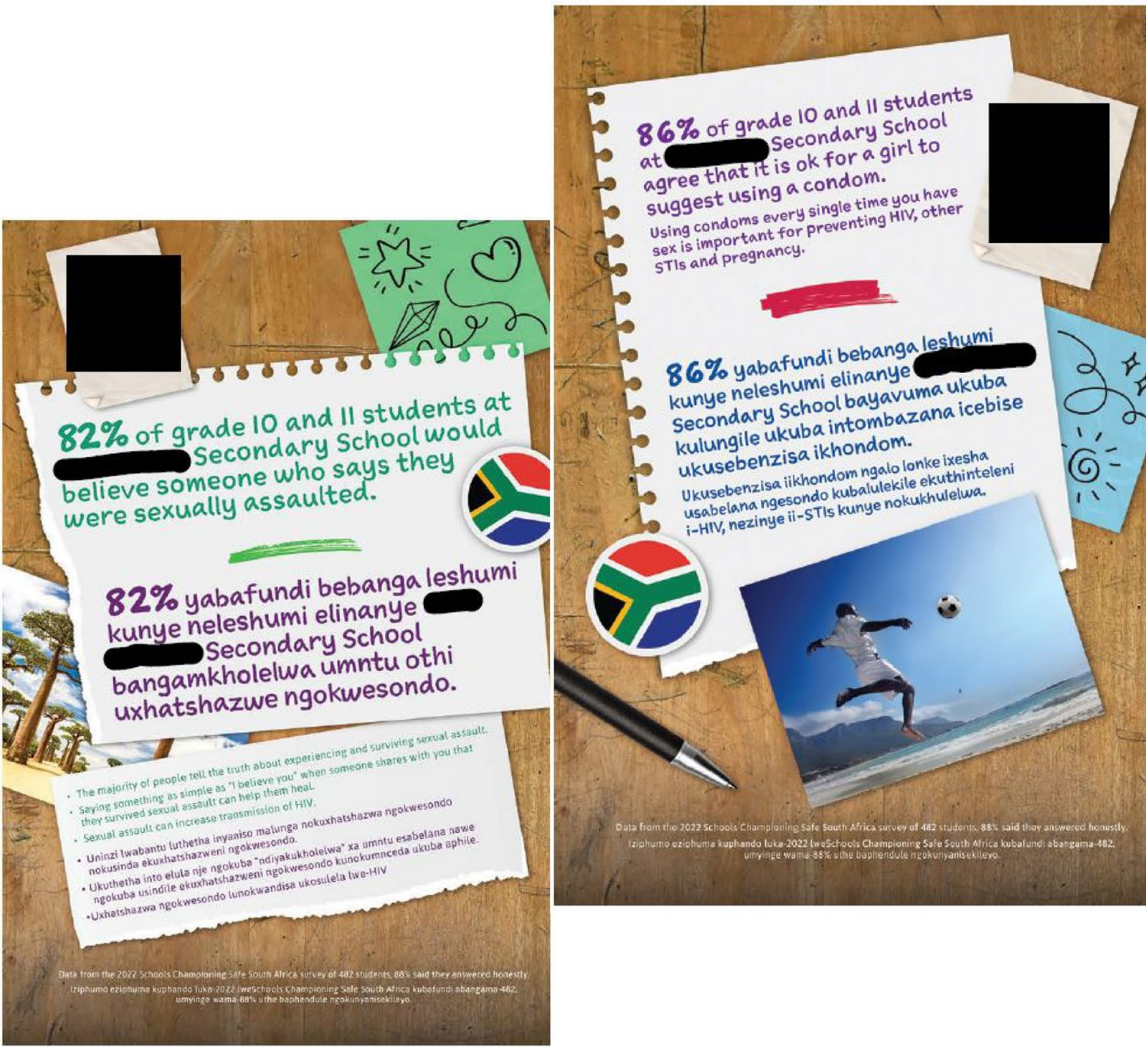
Examples of social norms posters featured at schools

For the first part of the intervention, the social norms marketing poster campaign, we specifically chose a low-tech approach (rather than apps, websites, other media) for South Africa with future sustainability and scalability in mind. The social norms poster campaign was generated from the school-specific data from boys, their student peers, and their teachers. Each poster targeted identified problem behaviors and/or core misperceptions in community norms relating to violence and HIV risk behavior for the school. In total, 12 posters were deployed covering the following themes: linkages between HIV and sexual violence, norms around violence, norms around relationship expectations in relation to intimacy, norms and behaviors around condom use, expectations around teacher involvement in addressing violence, norms about active bystander intervention including methods for effective active bystander intervention, norms around behaviors and gender equity in relationships, and gaining sexual consent. The campaign was rolled out with 2 posters posted throughout school spaces for two weeks, after which a new set of 2 posters was posted to avoid message fatigue, until the campaign was completed. An example of a message displayed on a poster was: 76% of grade 10 and 11 students at this secondary school would respect someone who tried to stop sexual violence. Sexual violence includes any unwanted or forced sexual activity/behavior. While each set of posters featured core misperceptions unique specific to each school, the themes in the posters were consistent across schools. Refer to Fig. 1 which shows examples of social norms posters featured at schools.

Following the poster campaign, for the second part of the intervention which focused on behavior change, was delivered via two 1-hour sessions focused on translating the data presented on posters into healthy behavioral practices relating to prevention of intimate partner violence and HIV/STI risk behavior. These two 1-hour sessions were deployed as part of the school mandated curriculum on “Life Orientation” given to all students in South African public schools. These lessons were purposively sequenced after the posters in order to pick up on poster content and specifically address questions about the posters as well as align behavioral training with what appeared in poster content. Intervention facilitators were part of the research team and were specifically trained to deliver this intervention and had experience in teenage HIV and IPV interventions in South Africa. While the goal of the intervention is eventually to have teachers take over, to ensure consistent intervention implementation in the pilot trial, facilitators were part of the research team. Their facilitation of the interactive behavioral intervention sessions was guided by a manual that described the theory of the intervention and supported the consistent delivery of the core components of behavior change.

## Methods

### Study Design

Following the social ecological survey and integration of that data in the intervention content (described above), we launched the pilot randomized controlled trial in two high schools, one randomly allocated to receive the intervention and the other allocated to the control condition. Data on feasibility, acceptability, and preliminary efficacy were derived from this pilot randomized controlled trial (RCT) of *N* = 282 teenage boys, completed from 2020 to 2023. We enrolled 282 students, with 141 in the intervention school and 141 in the control school. To be eligible, adolescent participants needed to: identify as boys, be aged 15–17 years, and be enrolled as students at the high school. All participants also had to provide parental consent and provide informed assent. The *n* = 141 boys assigned to the intervention school also completed satisfaction assessments. While teachers were not the target of the intervention, they are critical to future rollout of the intervention (since they would likely take over facilitation of the lessons in the intervention). To be eligible, teachers needed to be teachers at the target schools that were involved in the study. We recruited *N* = 80 teachers in the participating schools to complete assessments at baseline and post-intervention, and for the *n* = 40 teachers at the intervention school, surveys to evaluate satisfaction with the intervention program. We secured written, informed consent for participation from teachers.

Boys enrolled into the pilot RCT completed a baseline survey and follow-up surveys at 1- and 6-months following the *Schools Championing Safe South Africa* intervention (ClinicalTrials.gov # NCT05869864). Participants in the control school received the experimental intervention after all participants in the pilot RCT completed follow-up surveys. All study procedures and protocols were approved by a human research ethics committee (Brown University Protocol 2104002978, South African Medical Research Council Protocol EC004-2/2023).

### Sampling for the Pilot RCT

**Fig. 2:**
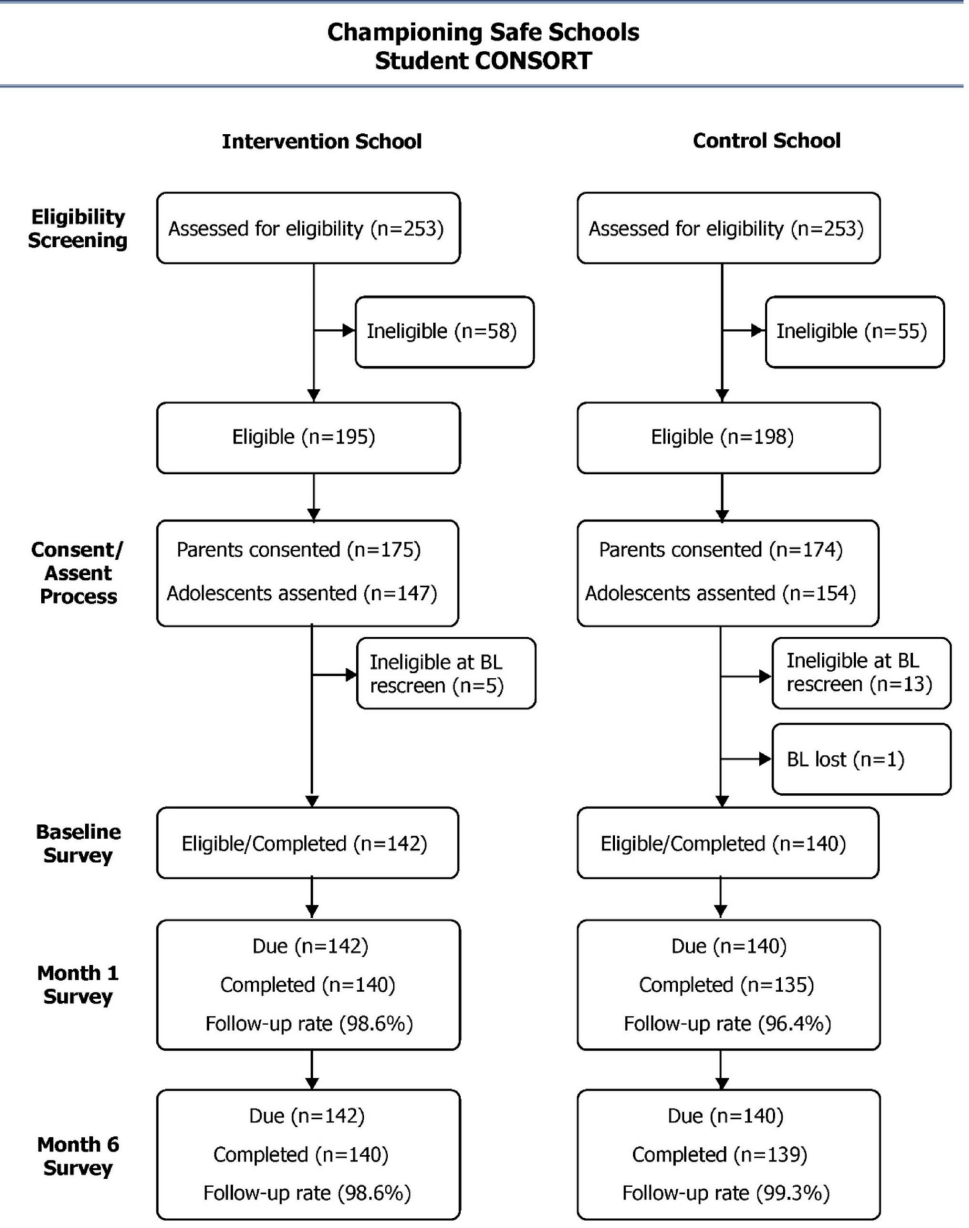
Championing Safe Schools Student Consort Diagram

All participants were recruited via a convenience sample from two typical public sector high schools in relatively poor peri-urban communities in South Africa. Trained study staff visited classrooms, briefly explained the purpose of the study, and handed out study information. After securing written informed assent and parent/guardian written consent, the study team conducted eligibility screening face-to-face with each student using a smartphone programmed with the eligibility criteria. Based on individual responses to the eligibility screening survey, the smartphone would inform the study staff member whether the individual was eligible or not. The recruitment and retention flow are shown in more detail in a CONSORT diagram (Fig. 2).

### Measures

To evaluate the primary outcomes of this study—acceptability and feasibility—a survey evaluating satisfaction of the content, format, and delivery of the intervention was administered to participants at the intervention school. For fidelity for the poster campaign, which was the first part of the intervention, we monitored exposure to the posters. For fidelity to the two behavioral intervention sessions delivered as Part 2 of the intervention, 20% of the sessions were evaluated by a neutral observer to determine whether the intervention was delivered as specified in the manual. We tracked receipt of intervention content including exposure to posters as well as receipt of Life Orientation sessions. We also tracked recruitment as well as retention of participants at each follow-up timepoint to evaluate the feasibility of a future larger trial. To evaluate preliminary efficacy of the intervention for participants in the pilot RCT, surveys were conducted at baseline, 1- and 6-months on a tablet, with sensitive questions issued via audio computer-assisted self-interviewing software. These surveys assessed: (1) HIV/STI testing and symptoms and sexual risk behavior data; (2) perpetration of sexual aggression, (3) norms and attitudes around sex, gender, and violence; (4) HIV knowledge; and (5) bystander intentions and behaviors. Students completed the survey in a classroom, facilitated by the study staff. The survey lasted approximately 60–80 min. Participants could toggle between their chosen language (English or isiXhosa) at any point throughout the survey. After completion of the baseline and 1- month surveys, teenagers and teachers received 70 Rand (equivalent to 3–5 USD) as reimbursement for their time. After completion of the 6-month survey teenagers and teachers received 90 Rand respectively (equivalent to 4–6 USD) as reimbursement for their time. Teenagers and teachers in the intervention school who completed acceptability surveys also received 50 Rand as reimbursement for their time.

#### Measures of Acceptability for Boys and Teachers

##### Client Satisfaction Survey

To evaluate intervention acceptability, a client satisfaction survey was administered to the participants (students and teachers) in the intervention school after both components of the intervention were fully deployed (the poster campaign as well as 2 Life Orientation sessions) [46]. The client satisfaction survey consisted of 8 multiple choice options and 6 free response items pertaining to the value, relevance, enjoyment, organization, and format of the intervention design and delivery. Most multiple-choice responses used various 4-point scales pertaining to feelings of satisfaction or dissatisfaction. Example items include “How would you rate the quality of the program you have received?” and “Has the information you received helped you to deal more effectively with issues important to you?” The free response questions asked about program location, length, staff, and reasons why it was difficult to attend the program sessions. Examples of free response questions include “Where would you like the program to be located next time?” and “How can we improve how we interact with you? Please be honest!” This survey was piloted in another HIV intervention study with teenagers of similar age in South Africa and performed well in that similar population [47]. 

#### Measures of Feasibility of the Intervention and Future Study

##### Fidelity

Fidelity pertains to the extent to which the key components of the intervention are implemented as intended. We examined intervention fidelity by evaluating how many of the target population were exposed to the posters. We also examined intervention fidelity by examining how well facilitators adhered to the manualized protocol of intervention delivery for the RCT. In the *Schools Championing Safe South Africa* intervention, delivery was guided by a manualized protocol that codified the core components of the intervention. We randomly selected 20% of the behavioral Life Orientation Lesson sessions to evaluate for fidelity. This resulted in the coding of 8 sessions total—four for Session One and four for Session Two. A neutral observer captured fidelity by live coding the delivery of the session, following a checklist of essential components of intervention delivery. The checklist assists the fidelity coder in evaluating how well facilitators adhered to the manualized protocol of the intervention. The fidelity coding form directly mapped onto the core behavior change components of the intervention and the standardized delivery guidelines that were laid out in the protocol.

The neutral observer rated the facilitator’s delivery skills and adherence to protocol using rankings from one (low demonstration of skills, < 25% of the time) to three (high demonstration of skills, > 75% of the time). In addition to marking if the core components of the intervention were covered, there were 11 delivery skills evaluated, including active engagement with participants; active listening; respectful, positive communication; guided discovery; warmth, concern, confidence, professionalism; empowerment of participants; building participant confidence; maintenance of appropriate pace of group discussion; and ending on a positive note. A ranking of 3 or above for adherence to the protocol and the majority of 11 delivery skills was viewed as “acceptable” for the pilot and would help identify future areas for facilitator training refinement and supervision should we proceed to a fully-powered efficacy trial.

##### Feasibility

We evaluated feasibility for implementing a future larger scale trial by examining recruitment and retention data focusing on the target population of boys. We also evaluated the feasibility of the intervention by looking at intervention attendance and completion of follow-up sessions.

#### Measures for Preliminary Efficacy

##### Lifetime Sexual Experiences and Condom Use

When first introduced within the survey, sexual acts (petting, oral, anal, vaginal sex) were defined for participants. Participants reported sexual experiences including how many times they engaged in oral sex, vaginal sex, and anal sex. They reported the number of people they have had vaginal or anal sex in their lifetime. Furthermore, participants were asked whether they used a condom since this is an important predictor for HIV and STI acquisition. We focused on condom use for penetrative sex, focusing on condom use at last vaginal sex and at last anal sex (yes/no) as well as consistent condom use. For consistent condom use, we focused on baseline versus 6-month analysis, because these two periods had a consistent look-back period of 3 months. Choice of questions for these HIV risk behaviors was informed by a review of HIV risk questions used by the US National Institutes’ of Health Teenage Medicine Trials Network for HIV/AIDS Interventions and have been previously tested in South African trials with similar populations [47]. 

##### Perpetration of Sexual Aggression

Perpetration of sexual violence and aggression was assessed via the Sexual Experiences Survey - Short Form Perpetration (SES-SFP) [48]. This 10-item measure captures frequency of attempted or completed sexual acts, tactics for each act, including, for example, via force, coercion, or incapacitation due to the administration of alcohol or drugs, and perception regarding whether the acts constituted rape. An example item includes: “I had oral sex with someone or had someone perform oral sex on me without their consent by threatening to physically harm them or someone close to them.” For each type of behavior—such as the unwanted oral sex item described previously—participants described the frequency (0, 1, 2, 3 + times in the past 12 months) and the tactics used (e.g. “threating to physically harm them or someone close to them”). Scoring yields the prevalence of individuals who have engaged in any perpetration behavior in the overall sample. To assess sexual violence perpetration, we assessed perpetration of any unwanted sexual contact (i.e., petting) or sexual intercourse (i.e., oral sex, vaginal sex, or anal sex) through a range of tactics (i.e., force, coercion, incapacitation via substances). Individuals were only counted once as a “yes” to perpetrating unwanted sexual contact regardless of how many times they may have perpetrated or whether they perpetrated sexual violence in multiple categories. The prevalence of perpetration in each category was also calculated (e.g., % of participants who engaged in unwanted oral sex one or more times, % of participants who engaged in unwanted anal sex one or more times). This measure has been specifically developed for teenage populations [49] and previously tested in previous South African trials with similar populations [47]. 

##### Bystander Intervention

Bystander intentions and behaviors were measured using an adapted Bystander Behavior Scale, with 8-items [50]. An example question is: “How likely are you to do something to try and stop what’s happening if a male peer or friend of yours is taking sexual advantage of a girl (like touching, kissing, having sex with) who is drunk, high from drugs, or passed out.” The questions are answered on a 5-point Likert scale, ranging from “Very unlikely” to “Very likely”. This measure was used in a similar intervention development study for teenagers in other settings [50]. 

### Sample Size and Statistical Analyses

The sample size for the RCT pilot was designed to examine whether there were promising directions for preventive behavior change but was not powered to examine efficacy. To evaluate preliminary efficacy, we analyzed data from the pilot RCT using an intent to treat analysis. Data relating to preliminary efficacy focused on measuring (1) reductions in actual or intended HIV risk behaviors; (2) reductions in IPV frequency and decreased endorsement of IPV supportive attitudes; and (3) increased proactive bystander intentions and/or behaviors.

Descriptive statistics were computed to assess sample characteristics and the distribution of study variables among participants in both arms of the pilot RCT. The direction of effects within the intervention and control arms for key outcomes from baseline to 6-month follow-up was evaluated using paired t*-*tests for continuous and ordinal variables converted to continuous form and McNemar’s Test was employed to assess within-arm changes over time for binary variables. Statistical significance was determined with a significance level set at 5% (*p* < 0.05) to assess significant change. Data analysis was performed using Stata version 18.

#### Signal of Behavior Change

The pilot trial was not powered to examine intervention effects. We were focused in this secondary analysis on examining signals for positive behavior change. All forms were checked for missing data in the field and during entry. Key variables were examined for skewness, variability, missing data, and outliers, with transformations to achieve normality, if needed. Behavioral outcomes were analyzed and compared within arms using McNemar’s Test for binary variables and paired t-test for continuous and ordinal variables (converted to continuous).

## Results

### Participant Characteristics

Participant demographics are summarized in Table 1. Almost all participants identified as Black African (99%) and isiXhosa speaking (97%). 69% reported engaging in at least one act of forced sexual aggression (petting, oral, vaginal or anal sex) at least once. The most common form of sexual aggression was perpetration of unwanted sexual contact (55%), followed by vaginal (38%), oral (32%) and anal sex (24%).

**Table 1:** Randomized control trial sociodemographic and behavioral characteristics

Variable	Control *N* = 140	Intervention *N* = 142	All *N* = 282
Age			
15 years	28 (20%)	29 (21%)	57 (20%)
16 years	66 (47%)	59 (42%)	125 (44%)
17 years	48 (39%)	52 (37%)	100 (35%)
Self-identified gender			
Boys	140 (100%)	142 (100%)	282 (100%)
Sex at birth			
Male	140 (99%)	140 (100%)	280 (99%)
Female	2 (1%)	0	2 (1%)
Self-identified ‘Race’^**a**^			
Black African	142 (100%)	136 (97%)	278 (99%)
Coloured	0	1 (1%)	1
Other	0	3 (2%)	3 (1%)
Language			
IsiXhosa	139 (98%)	134 (96%)	273 (97%)
Other	3 (2%)	6 (4%)	9 (3%)
Food Insecure^**b**^			
Yes	84 (59%)	83 (59%)	167 (59%)
No	49 (35%)	42 (30%)	91 (32%)
Unknown	9 (6%)	15 (11%)	24 (9%)
Self-reported HIV status			
Positive	6 (4%)	3 (2%)	9 (3%)
Negative	34 (24%)	42 (30%)	76 (27%)
Unknown	102 (72%)	95 (68%)	197 (70%)
Sexually active^c^			
Yes	129 (91%)	128 (91%)	257 (91%)
No	9 (6%)	10 (7%)	19 (7%)
Unknown	4 (3%)	2 (1%)	6 (2%)
Lifetime sexual activity by type			
Heavy petting	77 (54%)	75 (54%)	152 (54%)
Oral	61 (43%)	50 (36%)	111 (39%)
Anal	50 (35%)	45 (32%)	95 (34%)
Vaginal	118 (83%)	113 (81%)	231 (82%)
**Sexual Partners past 3 months**			
None	20 (17%)	12 (10%)	32 (13%)
1–2 partners	68 (58%)	72 (60%)	140 (59%)
3 + partners	29 (25%)	37 (31%)	66 (28%)
Sexual partners lifetime			
None	16 (12%)	17 (13%)	33 (13%)
1–2 partners	32 (25%)	25 (19%)	57 (22%)
3 + partners	82 (63%)	92 (69%)	174 (66%)
Ever used a condom^**d**^			
Yes	79 (66%)	80 (66%)	159 (66%)
No	38 (32%)	38 (31%)	76 (32%)
Condom use past 3 months			
Always or most times	54 (55%)	47 (43%)	101 (49%)
Sometimes	26 (27%)	41 (38%)	67 (32%)
Never	15 (15%)	18 (17%)	33 (16%)
Sexual Violence Perpetration: Completed one or more of these acts during past 12 months			
Overall prevalence	88 (65%)	100 (73%)	188 (69%)
Heavy Petting	83 (59%)	71 (51%)	154 (55%)
Oral	52 (37%)	37 (26%)	89 (32%)
Vaginal	60 (42%)	46 (33%)	106 (38%)
Anal	38 (27%)	31 (22%)	69 (24%)
Sexual Violence Perpetration: Attempted one or more of these acts during past 12 months			
Overall prevalence	54 (40%)	65 (49%)	121 (46%)
Oral	42 (30%)	37 (26%)	79 (28%)
Vaginal	44 (31%)	37 (26%)	81 (29%)
Anal	41 (29%)	28 (20%)	69 (24%)

^a^Race is self-identified and we use categories as defined in the South African census. We see race as a social construct. The apartheid system coined the categories of ’White’, ‘Black’, ‘Indian’ and ‘Coloured’ to achieve racial stratification needed to effect discriminatory practice. The use of these terms here does not legitimize their validity or biological plausibility but is rather intended to foreground the way discrimination was deployed by previous governments to determine health opportunities for all South Africa—on a discriminatory basis.

^b^“In the past four weeks, how often was there no food to eat of any kind in your house because of lack of money?”

^c^Sexually active was defined as ever having engaged in heavy petting, oral, vaginal and/or anal sex.

^d^Male and female condom included in question.

### Acceptability of the Intervention

#### Client Evaluations

All 140 teenage boys in the school allocated to the intervention arm received the intervention (100%) and responded to the client evaluations survey. Of these 140 teenage boys, all shared positive feedback regarding the intervention (Table 2).

**Table 2:** Students’ client evaluation survey results

Intervention quality	81% reported that they were mostly or very satisfied with the program.
	84.3% reported the quality of the program as “good” or “excellent”.
Intervention content	72.1% reported that the program “generally” or “definitely” gave them information that they wanted. Students wanted more information about STIs, sexual intercourse, more details on how to protect themselves from diseases.
	93.5% reported that the content in the intervention met “most” or “almost all” of their needs.
	77.8% reported that the intervention helped them to deal with issues that were important to them.
	66.4% noted that they were “mostly” or “very” satisfied with the amount of information they had in the program.
Intervention setting	73.1% reported they were “mostly” or “very” satisfied this took place in school; those who were less satisfied suggested the program take place in the community or on social media.
Intervention length	51.4% noted the program length was just right, with 30.7% wanting the program to be longer.
Other	88.6% reported they would come back to the program or want to see the program run again.
	70% would recommend the program to friends.

Out of 40 teacher participants in the intervention school, there was a 100% response rate. Of the 40 teachers who answered the client evaluation survey, all shared positive feedback regarding the intervention (Table 3).

**Table 3:** Teachers’ client evaluation survey results

Intervention quality	75% noted they were overall, “mostly satisfied” or “very satisfied” with the program.
	82.5% reported the quality of the program as “good” or “excellent”.
Intervention content	85.0% reported that the intervention helped them to deal with issues that were important to them.
	82.5% noted that they were “mostly” or “very” satisfied with the amount of information in the program.
Intervention length	45.0% noted the program length was just right, with 22.5% wanting the program to be longer.
Other	92.5% reported they would come back to the program or want to see the program run again.
	72.5% would recommend the program to other teachers.

### Feasibility of the Intervention

#### Fidelity to the Intervention

Of the 140 participants in the intervention arm, there was excellent exposure to the poster campaign. Between 96.4% and 99.3% of participants saw each of the posters. Of the 141 boys in the intervention group 112 reported attending the first Life Orientation lesson (79%), while 106 reported attending the second Life Orientation lesson (75%); attendance rates may have been higher, but some participants selected ‘prefer not to answer’ for attendance questions and therefore are not considered in the attendance category.

#### Feasibility of a Future Trial

We examined the feasibility of a future trial by examining recruitment and retention data.

Of the 506 total students assessed for eligibility, 282 were enrolled in the trial; 56% were found to be ineligible or didn’t provide parental and/or teen consent. Of the 80 total teachers assessed for eligibility, 80 (100%) agreed to be in the study. For retention, rates were excellent for students. At the 1-month follow-up timepoint, 98% were retained (275/282). At the 6-month follow-up timepoint, 99% were retained (279/282). Similarly, retention rates were excellent for teachers. At the 1-month follow-up timepoint, 100% of teachers were retained (80/80). At the 6-month follow-up timepoint, 95% were retained (76/80) (Fig. 2).

### Preliminary Efficacy of the Intervention

Regarding lifetime condom use, there were no significant changes in either the intervention or control groups between one-month and six-month follow up timepoints. Additionally, there were no significant changes in consistent condom use for both intervention and control groups across all time points (Table 4).

**Table 4:** Change in behavioral outcomes by study arm and timepoint

Variable	Baseline	Follow-up	Difference	*P*-value
Consistent condom use (Intervention), Mean (95% CI)				
Baseline vs. Month 6	2.48 (2.24; 0.72)	2.33 (2.12; 2.55)	0.14 (-0.09; 0.38) *	0.084
Consistent condom use (Control), Mean (95% CI)				
Baseline vs. Month 6	2.28 (2.00; 2.57)	2.22 (1.92; 2.51)	0.07 (-0.26; 0.39) *	0.684
Lifetime condom use (Intervention),** % (95% CI)**				
Month 1 vs. Month 6	67 (57; 76)	69 (60; 78)	-6 (-19; 8)	0.084
Lifetime condom use (Control), % (95% CI)				
Month 1 vs. Month 6	66 (56; 75)	71 (61; 80)	-1 (-14;11)	0.835
Any completed act of sexual perpetration—1 or more acts of forced touching, oral sex, anal sex, and/or vaginal sex (Intervention)), % (95% CI)				
Baseline vs. Month 1	71 (61; 80)	55 (44; 66)	15 (4; 26)	**0.004**
Baseline vs. Month 6	72 (62; 81)	68 (58; 77)	4 (-6; 14)	0.353
Any completed act of sexual perpetration—1 or more acts of forced touching, oral sex, anal sex, and/or vaginal sex (Control)), % (95% CI)				
Baseline vs. Month 1	63 (53; 73)	58 (47; 69)	4 (-8; 16)	0.446
Baseline vs. Month 6	61 (50; 72)	55 (44; 67)	6 (-7; 19)	0.345
Any attempted act of sexual perpetration—1 or more acts of attempted forced oral sex, anal sex, and/or vaginal sex (Intervention)), % (95% CI)				
Baseline vs. Month 1	49 (40; 58)	25 (17; 33)	22 (11; 32)	**< 0.001**
Baseline vs. Month 6	47 (40; 58)	43 (35; 54)	5 (-8; 17)	0.446
Any attempted act of sexual perpetration—1 or more acts of attempted forced oral sex, anal sex, and/or vaginal sex (Control)), % (95% CI)				
Baseline vs. Month 1	36 (27; 45)	27 (19; 36)	9 (-1; 19)	0.068
Baseline vs. Month 6	33 (27; 45)	26 (19; 35)	9 (-4; 21)	0.14
Bystander intentions (Intervention), Mean (95% CI)				
Baseline vs. Month 1	11.81 (9.89; 13.74)	11.77 (9.90; 13.64)	0.043 (-1.8; 1.93) *	0.964
Baseline vs. Month 6	12.42 (10.28; 14.56)	13.76 (11.60; 15.91)	-1.34 (-3.20; 0.53) *	0.157
Bystander intentions (Control), Mean (95% CI)				
Baseline vs. Month 1	12.94 (10.96; 14.92)	13.65 (11.51; 15.80)	-0.71 (-2.63; 1.20) *	0.463
Baseline vs. Month 6	13.01 (10.98; 15.04)	12.88 (10.68; 15.09)	0.13 (-2.10; 2.35) *	0.91

In the intervention school, we measured any completed acts of sexual perpetration (forced touching, oral sex, anal sex, and/or vaginal sex 1 or more times). Engagement in any type of sexual perpetration act significantly decreased from 71% (95% CI: 61%, 80%) at baseline to 55% (95% CI: 44%, 66%) at one-month follow-up, with a difference of 15% (95% CI: 4%, 26%; *p* = 0.004). However, by the six-month follow-up timepoint, this change was not significant (72–68%; *p* = 0.353). The control group showed no significant changes in any acts of completed sexual perpetration over time (Table 4). We also examined engagement in any attempted acts of sexual perpetration (attempted acts were defined as attempted but uncompleted acts of forced touching, oral sex, anal sex, and/or vaginal sex 1 or more times). The intervention group exhibited a significant reduction when we examined engagement in any act of attempted sexual perpetration, from 49% (95% CI: 40%, 58%) at baseline to 25% (95% CI: 17%, 33%) at one-month follow up with a proportion difference of 22% (95% CI: 11%, 32%; *p* < 0.001). This effect was not sustained at six-month follow up (47–43%; *p* = 0.446). The control group did not show significant changes in the number of acts of attempted perpetration across the time points (Table 4).

Finally, we examined intentions to be an active bystander to stop violence. There were no significant changes in this outcome. Similarly, there were no significant changes in consistent condom use for either the intervention and control groups across all time points (Table 4).

## Discussion

There is a gap in intervention science with few programs that meet the needs of adolescents who perpetrate, or who are at high risk for perpetrating intimate partner violence. Developing intervention science with approaches that align with the life stage and developmental needs of adolescents is especially vital in the South African setting where there are intersecting epidemics of both IPV and HIV. In this pilot trial, we were able to show that the intervention *Schools Championing Safe South Africa* was highly acceptable to adolescent boys who ranked the intervention quality and setting highly. The acceptability of the intervention is an important finding in and of itself, given that perpetration of violence is underreported and a difficult topic to discuss. It is promising that boys who engaged in violence and/or were at high risk for perpetrating violence liked the intervention; this population can be difficult to engage in such a difficult, stigmatized, and sensitive topic. While most intervention participants liked the intervention content, they wanted more information; as we prepare for the next stage of research, we will explore further what sort of intervention content needed more details. The majority of intervention participants felt the program length was just right or wanted it to be longer. Similarly, the majority of intervention participants would come to the program, want to see it run again, or would recommend to friends. The feasibility of running a larger trial was also high—with excellent fidelity to the intervention content by intervention facilitators as well as excellent retention rates for a trial with minors and covering sensitive content. This is a very positive finding given the challenges of addressing IPV issues with boys and the many failures reported in the literature. Our belief is that the use of normative feedback, which teaches boys about their peer group, serves to lower defensiveness, and foster engagement and curiosity, in addition to providing ‘good news’ and that the positive evaluations offer strong support for using a norms correction with boys in violence prevention trainings. This potential of this specific theoretical approach to address violence is supported by a review of the social norms literature that was published recently on the utility of this approach in violence prevention [51]. 

While the primary aim of this study was to examine acceptability and feasibility, we also examined direction of behavior change. Findings were also positive in this regard, showing strong signals of positive change for reducing both completed and attempted acts of IPV perpetration one month after the intervention adding to the nascent literature on interventions that address perpetration of sexual violence. In fact, in a meta-analysis of 20 trials involving over 37,000 adolescents showed effective interventions were few. Furthermore, the review indicated that programs delivered in schools and targeting adolescents in our target age range yielded significantly larger effects when compared to programs that were either delivered outside of schools or targeted adolescents under 15 years of age [52]. 

While the promising signals in our study disappeared at the second follow up timepoint, this is not uncommon in other studies on prevention of violence [53]. This may have been due to limitations of the study. While we did not see behavior signals for HIV behavioral risk reduction or bystander intentions, this may have been due to the small sample. In a fully powered efficacy trial, we would want to examine HIV behavioral risk reduction in a more expansive way including delay of sex, condom intentions, as well as biological measures (e.g. HIV testing). We also plan to expand our measurement of bystander intentions to also include bystander actions. Despite these limitations, developing intervention science to concomitantly address HIV along with violence using a theoretically grounded intervention using social norms is a new addition to the intervention science [51]. Another explanation for why promising signals of behavior change disappeared at the second follow up is due to the issue of rebound. Other evaluations of attitude and behavior change interventions have similarly found that after a reduction in the risk behavior, a rebound is observed [53, 54]. To eliminate such a rebound, we want to further explore whether the intervention needs to be longer or involve interactive reinforcers, also discussed in literature on sexual violence prevention [55, 56]. This could be accomplished by augmenting and reinforcing the intervention in a variety of ways: extending the time that posters are displayed in schools; adding a life-orientation session that re-caps and provides additional practice of the important behavioral skills training needed to prevent violence and HIV infection/acquisition; supplementing the intervention by encouraging teachers and school staff to talk about the lessons; training peer leaders to talk about and disseminate the study goals/lessons; and booster workshops designed to deepen cognitive changes relating to motivation to change as well as behavioral practice to sustain change; [56] instituting ways for students to anonymously report positive actions and then publicizing these stories; and finally implementing complementary interventions in other layers of the social ecology, such as community level or organizational level interventions [57]. 

We think that some of the positive signals of change in our intervention relate to the unusual aspect of combining a social norms media campaign with the in-person intervention sessions focused on behavior change; thus some of our considerations for augmenting the intervention focus on ensuring that behavioral components of the intervention remain as an important complement to the social norms aspects of the intervention. While many of these possible changes align with factors that were identified in a meta-review of successful adolescent HIV behavioral interventions [58], they need more exploration in relation to violence preventive interventions where knowledge of how to sustain violence prevention over time is less developed in the scientific literature [59]. In summary, we report here a number of positive outcomes: an actual reduction in violence committed by the experimental group, subject to a rebound commonly reported in the literature, and a very positive response of the intended audience and teachers to the intervention. That this intervention shows some promise, and with an age- and developmentally-appropriate approach for this group of adolescents indicates that further testing is needed to examine the efficacy of the intervention.

## Data Availability

Authors are open to sharing data and study materials.
